# EEG Bands of Wakeful Rest, Slow-Wave and Rapid-Eye-Movement Sleep at Different Brain Areas in Rats

**DOI:** 10.3389/fncom.2016.00079

**Published:** 2016-08-03

**Authors:** Wei Jing, Yanran Wang, Guangzhan Fang, Mingming Chen, Miaomiao Xue, Daqing Guo, Dezhong Yao, Yang Xia

**Affiliations:** ^1^Key Laboratory for NeuroInformation of Ministry of Education, Center for Information in BioMedicine, School of Life Science and Technology, University of Electronic Science and Technology of ChinaChengdu, China; ^2^Department of Herpetology, Chengdu Institute of Biology, Chinese Academy of SciencesChengdu, China

**Keywords:** factor analysis, frequency band, SWS, REM sleep, wakeful rest, power spectra, rat

## Abstract

Accumulating evidence reveals that neuronal oscillations with various frequency bands in the brain have different physiological functions. However, the frequency band divisions in rats were typically based on empirical spectral distribution from limited channels information. In the present study, functionally relevant frequency bands across vigilance states and brain regions were identified using factor analysis based on 9 channels EEG signals recorded from multiple brain areas in rats. We found that frequency band divisions varied both across vigilance states and brain regions. In particular, theta oscillations during REM sleep were subdivided into two bands, 5–7 and 8–11 Hz corresponding to the tonic and phasic stages, respectively. The spindle activities of SWS were different along the anterior-posterior axis, lower oscillations (~16 Hz) in frontal regions and higher in parietal (~21 Hz). The delta and theta activities co-varied in the visual and auditory cortex during wakeful rest. In addition, power spectra of beta oscillations were significantly decreased in association cortex during REM sleep compared with wakeful rest. These results provide us some new insights into understand the brain oscillations across vigilance states, and also indicate that the spatial factor should not be ignored when considering the frequency band divisions in rats.

## Introduction

Neuronal oscillations in cortical networks are supposed to be essential for information communication, thereby underlying the fundamental brain functions (Basar et al., 2001; Buzsaki and Draguhn, 2004). What's more, it has been recognized that neural oscillations in the brain are often separated into several oscillatory bands, and each frequency band may originate in different cortical and subcortical structures, such as delta and spindle oscillations originate from the thalamo-cortical network (Dossi et al., 1992; Steriade et al., 1993), while theta oscillations are the most prominent in septo-hippocampal system (Nunez et al., 1987; Leung and Yim, 1991). Accumulating evidence suggests that specific brain states related to kinds of cognitive functions, such as perception, attention, and memory (Klimesch, 1999; Basar et al., 2001; Thut and Miniussi, 2009), are tightly associated with these oscillatory activities (Klimesch, 1999; Engel et al., 2001; Nicolelis et al., 2008).

In humans, the electroencephalogram (EEG) is widely used in the physiological and pathological studies (Babiloni et al., 2007; Basar and Güntekin, 2008; Lomas et al., 2015), in which EEG is often manually subdivided into broad frequency bands through visual inspection (Nunez and Cutillo, 1995). Similarly, in animals, frequency bands are always divided arbitrarily and determined empirically, based on the presumed homologous frequency bands in humans (Lancel and Kerkhof, 1989; Trampus et al., 1990; Grasing and Szeto, 1992). To our knowledge, only few studies determine the relevant functional frequency bands with multivariate statistical methods in humans (Klimesch, 1999; Corsi-Cabrera et al., 2000), rats (Corsi-Cabrera et al., 2001) and frogs (Fang et al., 2012). Actually, previous studies show that the frequency bands acquired from principal component analysis (PCA) are more consistent with the underlying physiological mechanisms in rats (Corsi-Cabrera et al., 2001). Note that electrophysiological oscillations are sensitive to recording sites (Siapas and Wilson, 1998; Klimesch, 1999; Sirota et al., 2003), indicating the possible involvement of different brain oscillatory generators (Mitchell et al., 2008; Young and McNaughton, 2009; Fang et al., 2010; Timofeev and Chauvette, 2013). Therefore, considering the spatial factor in the division of frequency bands would be more fulfilling (Klimesch, 1999).

Slow waves and spindle activities are the main landmarks of slow-wave sleep (SWS) (Steriade et al., 1993). In rats, studies of slow oscillations have been frequently restricted to 1–4 Hz, while frequency ranges of sleep spindles are shown in a wider distribution, from 6–12 to 14–20 Hz (Siapas and Wilson, 1998; Eschenko et al., 2006; Mölle et al., 2006; Johnson et al., 2010; Peyrache et al., 2011). During wakefulness and rapid-eye-movement sleep (REMS), a series of neurotransmitter systems abolish sleep spindles and slow waves (Steriade et al., 1993), which are replaced by “desynchronized” low-amplitude activities of beta and gamma from 10–15 to 80–120 Hz (Corsi-Cabrera et al., 2001; Brown et al., 2012). Unlike the division of theta and alpha bands in humans, alpha band is replaced by a wider range of the theta band (usually 4–12 Hz) in rats (Corsi-Cabrera et al., 2001; Buzsáki, 2006).

Factor analysis, a multivariate statistical method, facilitates identification of electrophysiological patterns neglected by visual inspection (Fang et al., 2012). It was hypothesized that the bands extracted by factor analysis would fit better the functional oscillations within the specific brain regions during specific behavior states. With this purpose, we concentrated on several brain regions of rats, using the factor analysis method, to extract the covariance of different frequencies across vigilance states, including wakeful rest (WR), SWS, and REMS. The results showed that the band divisions differ across vigilance states and brain regions. Unlike the traditional frequency band divisions, which here are more in accordance with the potential functional oscillatory activities.

## Materials and methods

### Animals

Thirteen male Wistar rats (weighting 260–290 g) were used in this experiment. Before the surgery, rats were housed in small groups with food and water *ad libitum* and were maintained on a 12 h light/dark cycle (white lights on at 8:00). All experiments were approved by the Ethical Committee on Animal Experimentation of the University of Electronic Science and Technology of China (UESTC).

### Surgery

**Figure 1:**
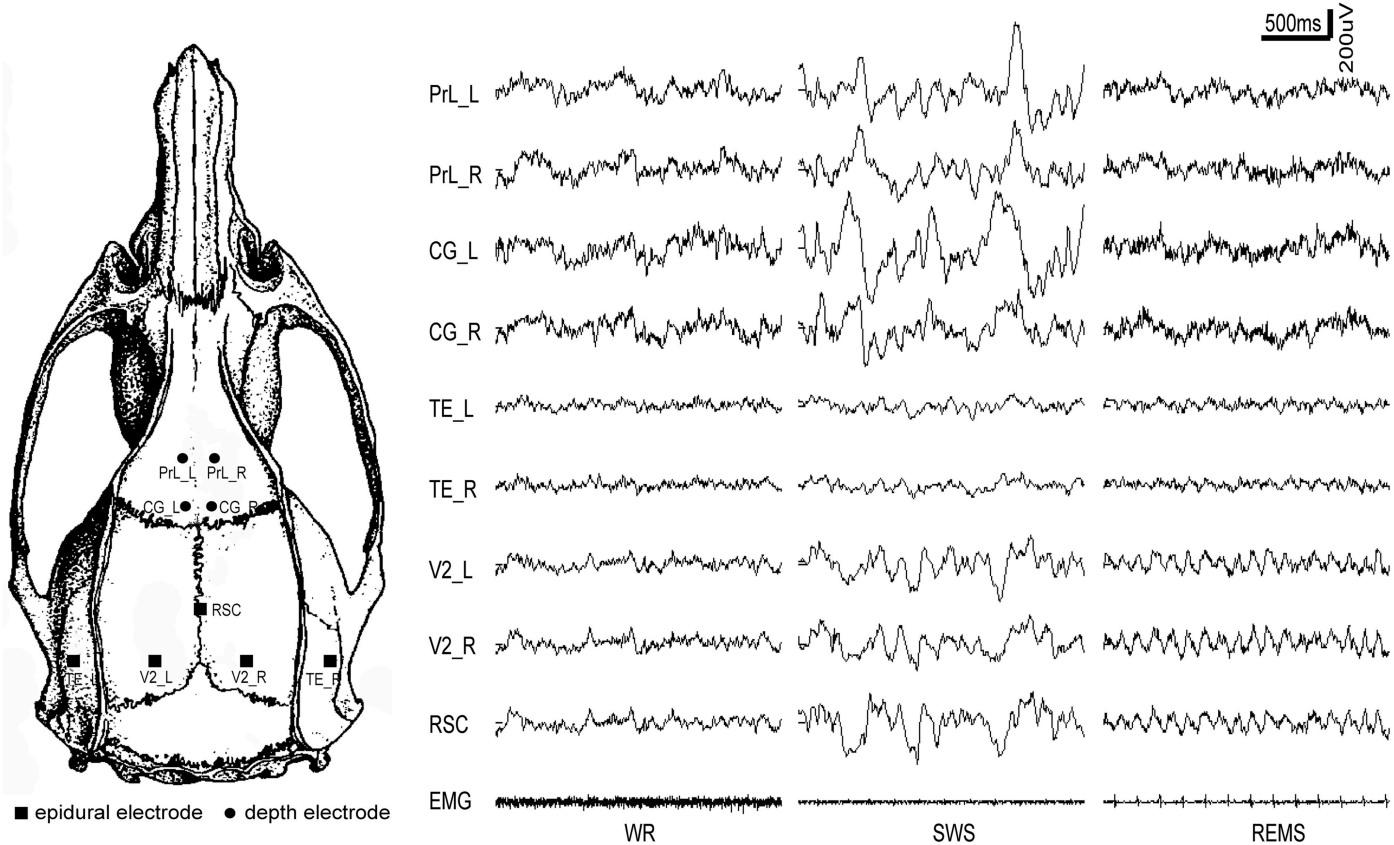
**The distribution of intracranial electrodes and the corresponding EEG tracing for each electrode under different vigilance states**. PrL, prelimbic cortex; CG, cingulate cortex; RSC, retrosplenial cortex; V2, secondary visual cortex; TE, temporal cortex (Auditory/temporal association cortex); L, left; R, right; WR, SWS, and REMS, the three vigilance states.

Chronic electrode implantation was performed under general anesthesia (sodium pentobarbital 60 mg/kg body weight, i.p.), complemented with 0.6 ml atropine sulfate (0.5 mg/ml, s.c.) to prevent excessive secretion of the respiratory tract. Additional pentobarbital (15 mg/kg) was given intraperitoneally when required. Before and after the resection of the temporal muscle, local analgesia was administered with lignocaine (2%). All stereotactic coordinates were relative to bregma with the skull surface flat, according to Paxinos and Watson (2005). Each rat received five epidural cortical electrodes (stainless-steel screw; diameter, 500 μm) and four depth electrodes (insulated nichrome wires; diameter, 200 μm). Reference was set at the cerebellum. The coordinates of the electrodes and the 9-electrode montage with their typical EEG during three states (WR, SWS, and REMS) were shown in Table 1 and Figure 1, respectively. The temporal electrode implantation followed the procedure introduced by Meeren et al. (2001). Two electromyogram (EMG) electrodes were implanted bilaterally in the dorsal neck muscles. After the surgical procedure, penicillin G was used for anti-infection. All rats were given at least 2 weeks to recover before the recording sessions started.

**Table 1:** **Coordinates of the electrodes in rats**.

	**Paxino's atlas**		
**Region**	**A–P**	**M–L**	**D–V**
PrL	4.2	±0.8	3
CG	1.7	±0.7	2.6
RSC	−3.3	0	0
V2	−5.2	±2.4	0
TE	−5.2	±8	5
Rf.	−11	0	0

A–P, M–L, and D–V indicate anterior-posterior, medial-lateral, and dorsal-ventral directions, respectively. PrL, prelimbic cortex; CG, cingulate cortex; RSC, retrosplenial cortex; V2, secondary visual cortex; TE, temporal cortex (Auditory/temporal association cortex); Rf., reference.

### Recordings

Animals were habituated to the experimental environment 2 days prior to the recording. The connector on the rat skull was connected to a long recording lead, which allowed the subject to move freely in a homemade glass box (40 × 50 × 60 cm). Video recording was synchronized with the signal acquisition. Local field potential (LFP), electrocorticogram (ECoG) and EMG were recorded with a signal acquisition system (Chengyi, RM62160, China). Data were continuously recorded for 72 h (recording onset at 12:00). Bandpass filters were set between 0.16 and 100 Hz for EEG and between 8.3 and 500 Hz for EMG (50 Hz notch filter). The sample frequency was set at 1000 Hz. All recordings were stored on a hard disk (Lenovo Company, USA) for further analysis. All experiments were performed in a noise-attenuated room in which the environmental background noise was 32.2 ± 3.0 dB (mean ± SD). Other environmental variables (light: 12-h light/dark cycle [white lights on at 8:00]; food and water: *ad libitum* access; temperature: 25 ± 0.5 degree centigrade) were maintained. The experimenter entered the noise-attenuated room daily to clean cages and replace food and water at 12:00.

### Histological tests

To precisely determine the anatomical location of the depth electrodes, the animals were deeply anesthetized with chloral hydrate (300 mg/kg) after experiments. Then, the animals were perfused intracardially with saline followed by a 4% paraformaldehyde phosphate buffered solution. Brains were removed and stored in the paraformaldehyde phosphate buffer for 1 day and gradient dehydration with sucrose (15 and 30%) 2 days before sectioning. Serial coronal sections of 30 μm were cut on a freezing microtome, attached on Poly-L-Lysine-coated slides, and stained with ferric-chloride solution (Riboni et al., 1991). After the histological inspection, the rats with any electrode out of the designed anatomical location were excluded (3 rats), and data from the other 10 rats were included for further analyses (Figure S1).

### Data selection

To ensure that the rats were sufficiently adapted to the recording environment before obtaining data, only the data acquired on the third day (the last 24 h) were included in our analysis. Both SWS and REMS waves were selected from 8:00 to 12:00 (the last 4 of the 24 h, this period was rich in sleep activity). For each SWS and REMS segment, only state period >60 s, then the epoch was selected excluding the two sides of the data about 20 s (the epoch was selected as an integral multiple of 5 s). The 12:00 to 15:00 period was excluded because of the disturbance of the experimenter. If the selected data segments were less than the assumed 10 segments, the data from 15:00 to 20:00 were then included (from the 4th to the 8th h of the last 24 h). The wakeful rest data were chosen from the rest of the 17 h daily cycle, except from 8:00 to 15:00 (from the 4th to the 20th h of the last 24 h). For the wakeful rest data, only epochs during this state and more than 7 s were used to select the median 5 s segments for further analysis. The state selection was based on characteristics of EEG, MEG, and behaviors, which was systematically summarized in Table 2 (also see Figure 1).

**Table 2:** **Selection rules for data**.

**State**	**Electrical activity**		**Behaviors**	**Data size (one rat)**
	**EEG (LFP and ECoG)**	**EMG**		
WR	Mixed-frequency EEG activity	Relatively low and stable EMG activity	Standing or sitting quietly	Sixty segments (5 s per segments)
SWS	High-amplitude, low-frequency EEG activity	Low-level EMG activity	Lying or curl itself up	Ten segments (45–135 s per segments)
REMS	Sawtooth-pattern EEG activity	Flat EMG activity	Lying or curl itself up	Ten segments (50–140 s per segments)

### Data processing

Matlab (release 2013a) and SPSS software (release 19.0) were combined for the data processing. First, each segment of SWS and REMS was divided into 5-s epochs. Each epoch for the three states was band-pass filtered (0.5–34 Hz, to exclude potential high-frequency noise including muscle activities), down sampled (at 256 Hz) and de-trended (remove the linear trend). Power spectra with 1 Hz resolution were calculated by the Welch's method with the Hamming window. All the power spectra were first log-transformed and then averaged over brain areas, states and rats (one value for each brain area, state and rat) for further statistical analysis.

In this study, for each rat the log-transformed power spectra of each segment acquired from each electrode in SWS and REMS was averaged and the 60 epochs of WR were averaged in blocks of 6. Then 10 averaged values were computed for each state, electrode and rat. To compute frequency covariant with each other and to extract the frequency bands of each brain area across states, these 10 values were pooled for rats and brain areas for factor analysis (in each state, 200 values for PrL, CG, V2, and TE; 100 values for RSC).

Each brain area under each state was submitted to factor analysis with EEG frequencies as variables and the PCA as the extraction method. The following criteria were used: the Kaiser–Guttman criterion (eigenvalues higher than 1 for eigenvectors) combined with the scree slope method and frequency bands with loading factor above 0.5 were included. The varimax method was used for factor rotation. To further check the independence of factors, the promax method was also used. Although most of the correlations between factors reached a meaningful significance level, which reflects that the factors were correlated with one another (Figure S2), the results from the two methods were almost identical. Therefore, only the promax-based results are presented.

### Statistical analysis

In order to evaluate differences in power spectra, 3-way repeated measures ANOVA was used for within-subject variables (state, brain region, and frequency). Both main effects and interactions were examined. Simple or simple-simple effects were further applied when the interactions were significant. Partial η^2^ were employed for estimating of effect size of ANOVAs, and the values of 0.2, 0.5, and 0.8 were successively corresponding to small, medium, and large effect size (Cohen, 1992). For *post-hoc* multiple comparisons, the paired *t*-tests were used. The threshold of significance was set at a Bonferroni-adjusted *p*-value of 0.05.

## Results

### Frequency bands for different states and brain regions

To guarantee the suitability of our data for factor analysis and to ensure the reliability of the results, both Kaiser-Meyer-Olkin (KMO) test and Bartlett's test were performed. Table 3 showed the results of KMO and Bartlett's test. The KMO test showed the adequacy of sample size for all variables in the range from 0.89 to 0.97. Similarly, Bartlett's tests of sphericity were in the range from 4487.5 to 20181.68, and all of them were significant (*p* < 0.001).

**Table 3:** **KMO and Bartlett's test of five brain regions under the three vigilance states**.

	**KMO test**			**Bartlett's test**		
	**WR**	**SWS**	**REMS**	**WR**	**SWS**	**REMS**
PrL	0.91	0.95	0.93	8522.54	16242.33	14007.16
CG	0.91	0.93	0.93	8030.95	15171.37	13909.12
RSC	0.90	0.92	0.89	4487.50	6316.15	5663.79
V2	0.93	0.94	0.92	9131.12	15139.31	13312.74
TE	0.94	0.97	0.94	10176.72	20181.68	15074.94

Note that KMO scores should be over 0.8 to ensure the data robustness.

Factor analysis showed that during WR, four eigenvectors accounted 79.26, 77.41, and 81.65% of the total variance successively for PrL, CG and RSC; three eigenvectors explained 77.86 and 81.54% of the total variance for V2 and TE, respectively (Table 4). During SWS, four eigenvectors accounted 92.54, 89.94, and 93.04% of the total variance successively for CG, RSC, and V2; three eigenvectors accounted 91.66% of the total variance for PrL; two eigenvectors explained 95.32% of the total variance for TE (Table 4). During REMS, four eigenvectors explained 89.75, 88.81, and 87.94% of the total variance successively for PrL, CG, and V2; five eigenvectors accounted 86.48% of the total variance for RSC, and three eigenvectors explained 89.80% of the total variance for TE (Table 4).

**Table 4:** **Results of factor analysis on EEG power spectra from 1 to 32 Hz**.

	**Bands(Hz)**			**Cumulative %**		
	**WR**	**SWS**	**REMS**	**WR**	**SWS**	**REMS**
PrL	8–20	16–32	18–32	79.26	91.66	89.75
	22–32	1–6	8–11,14–17			
	1–4	7–14	1–5			
	5–7		5–7			
CG	8–21	17–32	12–32	77.41	92.54	88.81
	22–32	1–6	1–5			
	1–4	6–10	8–10,15–16			
	4–6	10–16	6–7			
RSC	21–32	9–21	18–27	81.65	89.94	86.48
	9–19	22–32	8–10,14–17			
	1–4	1–6	1–4			
	5–8	6–9	28–32			
			5–7			
V2	8–21	21–32	18–32	77.86	93.04	87.94
	22–32	10–20	8–11,13–18			
	1–6	1–6	1–4			
		6–8	5–7			
TE	8–20	9–32	1–15	81.54	95.32	89.80
	21–32	1–7,11	20–32			
	1–6		15–19			

**Figure 2:**
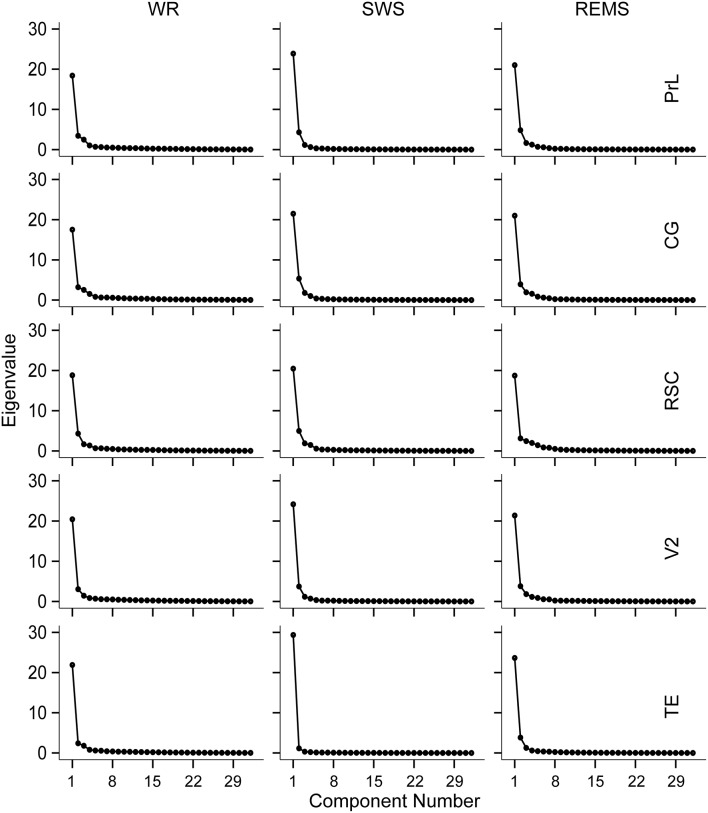
**Scree plots derived from the scree slope method for the five brain regions during WR, SWS, and REMS, respectively**.

**Figure 3:**
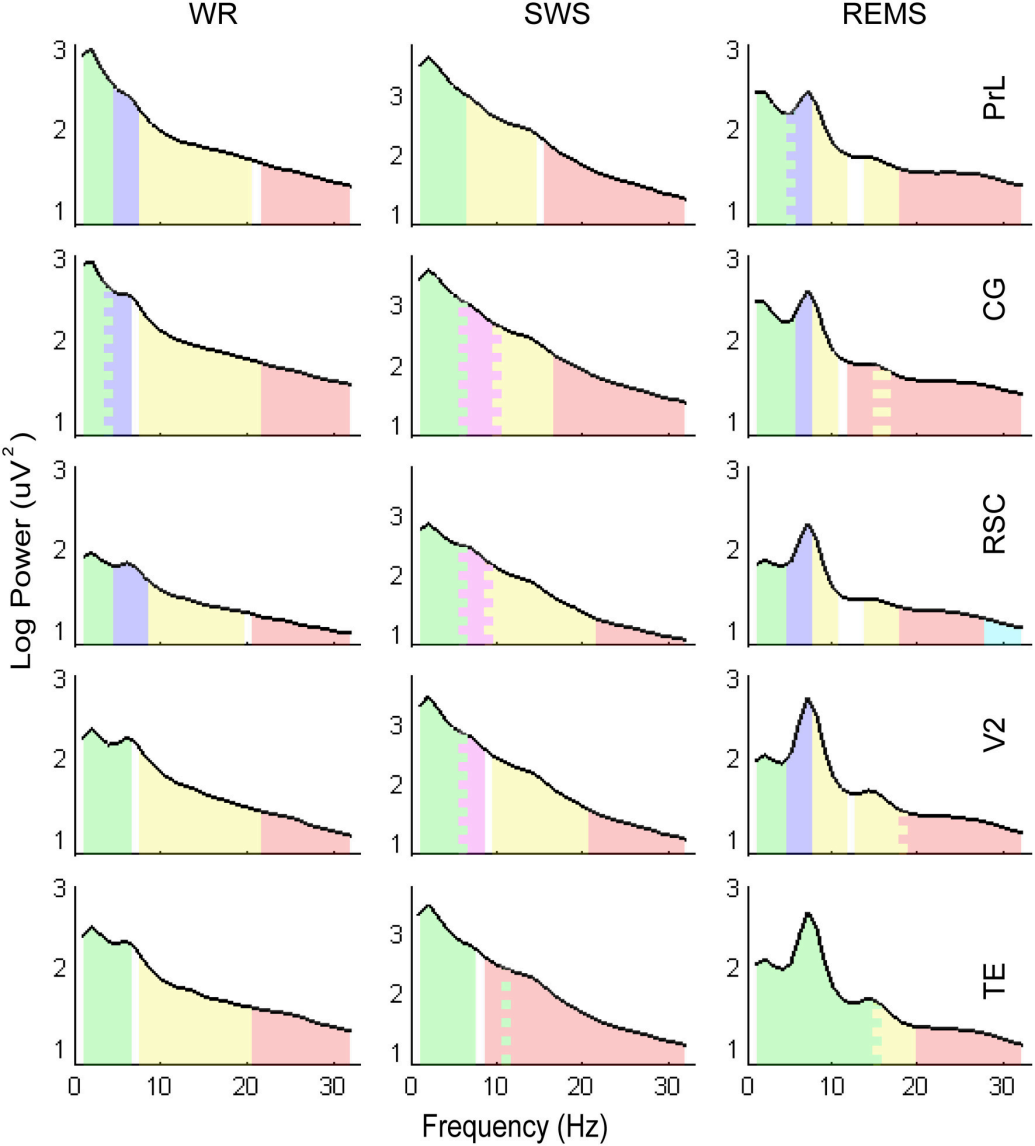
**Illustrations of the frequency bands and power spectra**. Log transformed EEG power spectra obtained from the mean of the entire group (*n* = 10). Colored areas represent the frequency bands clustered by factor analysis, corresponding to the band divisions in Table 4. The saw-tooth patterns indicate the overlaps of frequency bands.

Combining with the Kaiser–Guttman criterion and the scree slope method (Figure 2), during WR, a fast frequency band from 21–22 to 32 Hz and an intermediate band from 8–9 to 19–21 Hz were detected for all regions; two slow bands from 1 to 4 Hz and from 4–5 to 6–8 Hz were extracted for PrL, CG, and RSC, whereas, only one slow band from 1 to 6 Hz was identified for V2 and TE (Table 4 and Figure 3).

For SWS, a consistent slow band from 1 to 6 Hz was detected for PrL, CG, RSC, and V2, but a slow band from 1 to 7 Hz co-varying with frequency 11 Hz, was detected for TE; a fast frequency band from 16–22 to 32 Hz was extracted for PrL, CG, RSC, and V2; two intermediate bands from 6 to 8–10 Hz and from 9–10 to 16–21 Hz were identified for CG, RSC, and, V2, however, only one intermediate band from 7 to 14 Hz was extracted for PrL; there was no further subdivision of frequency band from 9 to 32 Hz for TE (Table 4 and Figure 3).

During REMS, two slow bands from 1 to 4–5 Hz and 5–6 to 7 Hz were detected for PrL, CG, RSC, and V2; a narrow band from 8 to 10–11 Hz that co-varied with higher frequencies from 13–15 to 16–18 Hz was also identified for PrL, CG, RSC, and V2; a fast band from 12–18 to 32 Hz was extracted for PrL, CG, and V2, whereas, two fast bands of 18–27 and 28–32 were identified for RSC, respectively; the division of frequency bands for TE was unique, including 1–15, 15–19, and 20–32 Hz (Table 4 and Figure 3).

### EEG power variations across states, brain regions, and frequencies

To make sure the power spectra of the selected EEG epochs during each vigilance state, we further evaluated differences in the power spectra. The results of the ANOVA revealed that all the main effects were significant for the factor “state” [*F*_(2, 18)_ = 913.174; *p* < 0.001, partial η^2^ = 0.990], the factor “brain region” [*F*_(2.237, 20.134)_ = 123.712; *p* < 0.001, partial η^2^ = 0.932], and the factor “frequency” [*F*_(2.490, 22.406)_ = 3108.309; *p* < 0.001, partial η^2^ = 0.997]. Additionally, all the interactive effects were also significant for “state ^*^ brain region” [*F*_(2.753, 24.778)_ = 33.634; *p* < 0.001, partial η^2^ = 0.789], “state ^*^ frequency” [*F*_(3.686, 33.177)_ = 317.453; *p* < 0.001, partial η^2^ = 0.972], “brain region ^*^ frequency” [*F*_(4.474, 40.266)_ = 56.639; *p* < 0.001, partial η^2^ = 0.863], and “state ^*^ brain region ^*^ frequency” [*F*_(5.824, 52.419)_ = 26.087; *p* < 0.001, partial η^2^ = 0.743]. Obviously, the three interactions were significant, so we performed simple-simple effect analysis.

**Figure 4:**
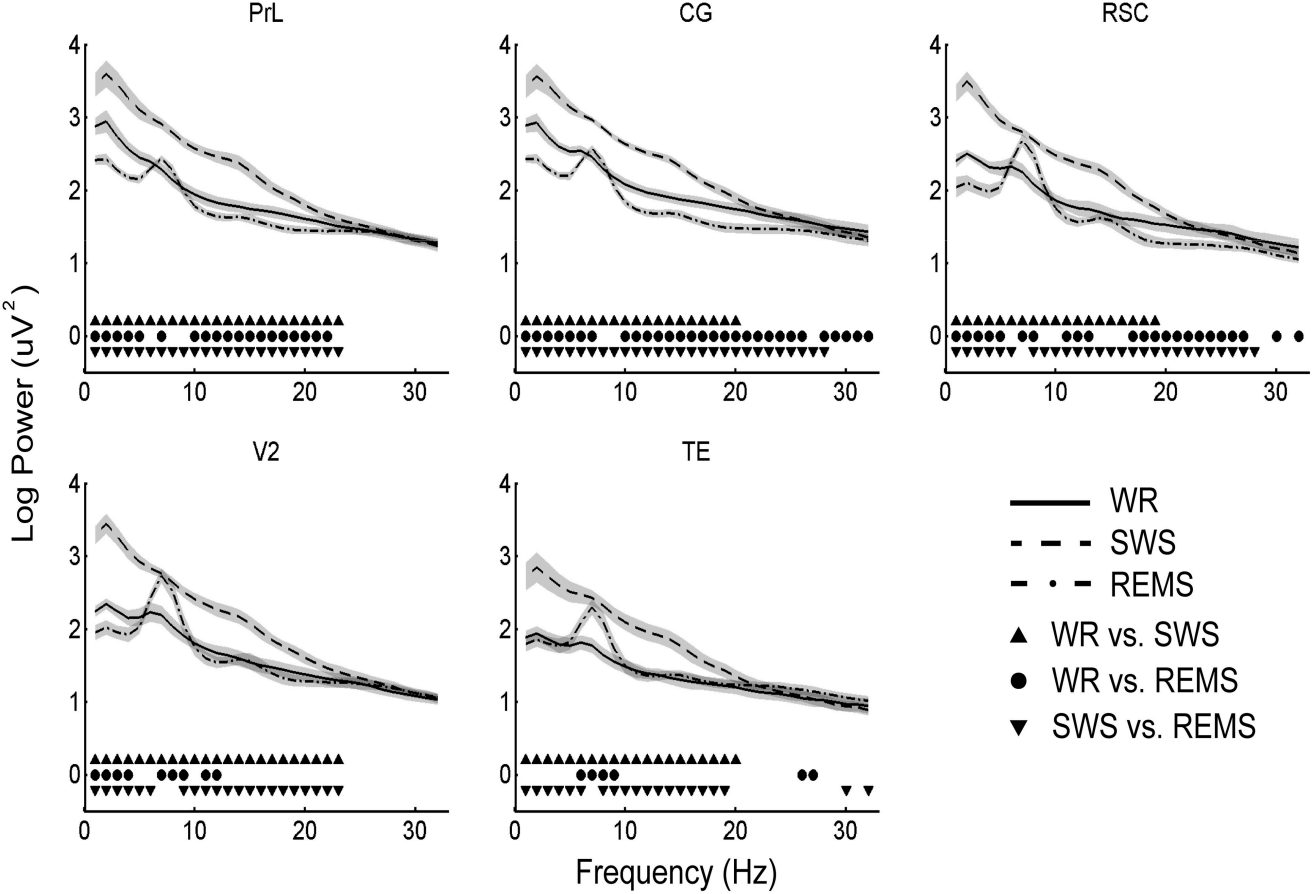
**Mean (*n* = 10) and standard deviation (shaded areas) of EEG power spectra during WR, SWS, and REMS for each brain region**. All the solid symbols indicate significant differences between states (*p* < 0.05, Bonferroni corrected).

Because EEG power spectra were especially susceptible by the reference electrode, we only focused on the factor “state.” Power spectra during SWS were significantly higher than that during WR for 1–23 Hz in PrL, for 1–20 Hz in CG, for 1–19 Hz in RSC, for 1–23 Hz in V2, and for 1–20 Hz in TE; power spectra during SWS were significantly higher than that during REMS for 1–23 Hz in PrL, for 1–28 Hz in CG, for 1–6 and 8–28 Hz in RSC, for 1–6 and 9–23 Hz in V2, and for 1–6 and 8–19 Hz in TE (significantly lower was also found in 30 and 32 Hz in TE); REMS showed significantly lower power than WR for 1–5 Hz and 10–22 Hz in PrL, for 1–6, 10–26, and 28–32 Hz in CG, for 1–5, 11–13, 17–27, 30, and 32 Hz in RSC, and for 1–4 and 11–12 Hz in V2, but higher than WR for 7 Hz in PrL and CG, for 7–8 Hz in RSC, for 7–9 Hz in V2, and for 6–9 and 26–27 Hz in TE (*p* < 0.05, Bonferroni corrected; Figure 4).

**Figure 5:**
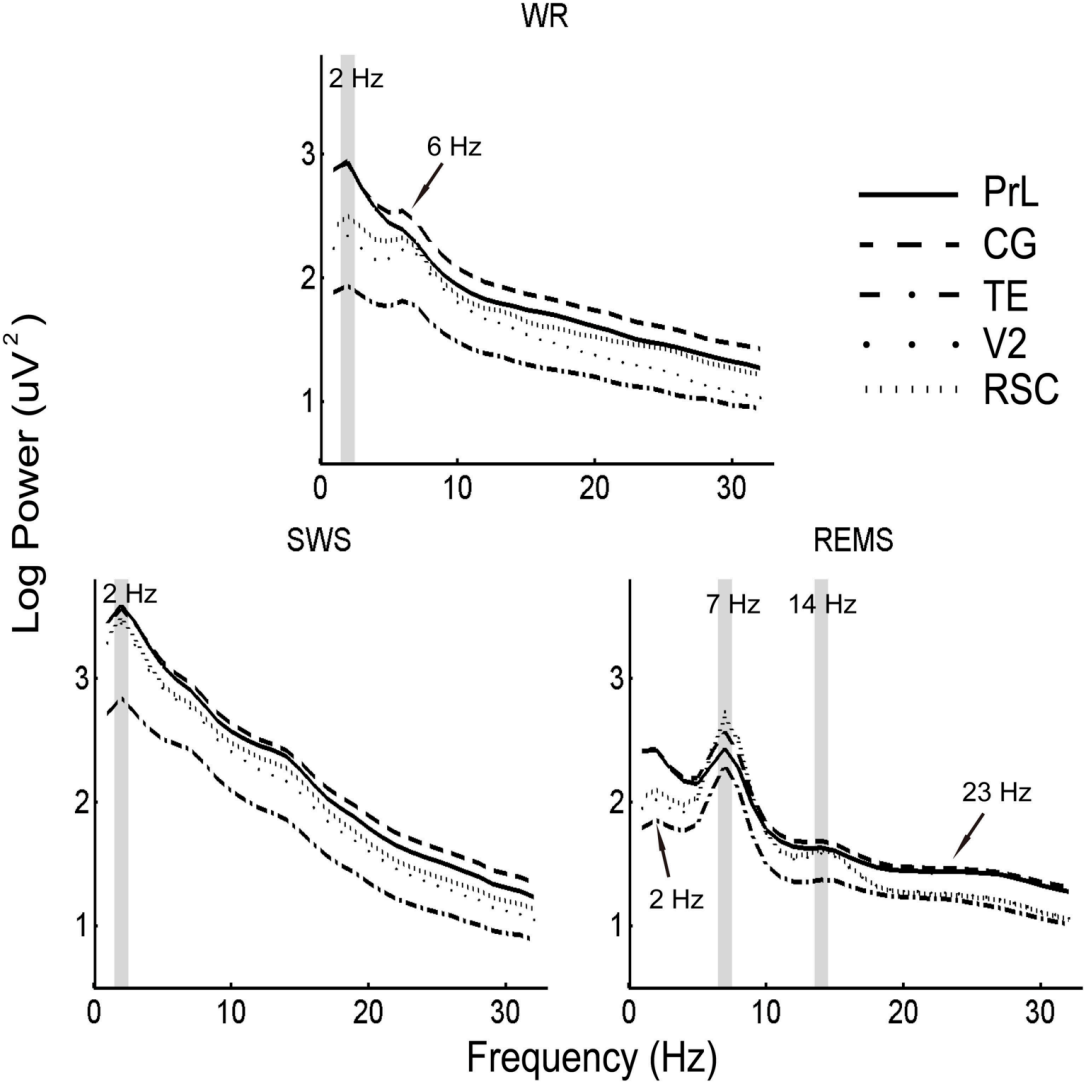
**Mean (*n* = 10) of EEG power spectra in the five brain areas during WR, SWS, and REMS**. Shaded areas denote coincident power peaks across brain areas, and arrows indicate the majority of peaks among regions.

To further reveal the distribution of power spectra across brain areas, the positive peaks in each region were calculated. Coincident slow oscillation frequency peaks occurred at 2 Hz during WR and SWS, and coincident frequency peaks occurred at 7 and 14 Hz during REMS. However, the 6 Hz peaks were found among regions (except PrL) during WR, the 2 Hz peaks were identified among regions (except CG) during REMS, and the 23 Hz peaks were detected among regions (except TE) during REMS (Figure 5).

**Figure 6:**
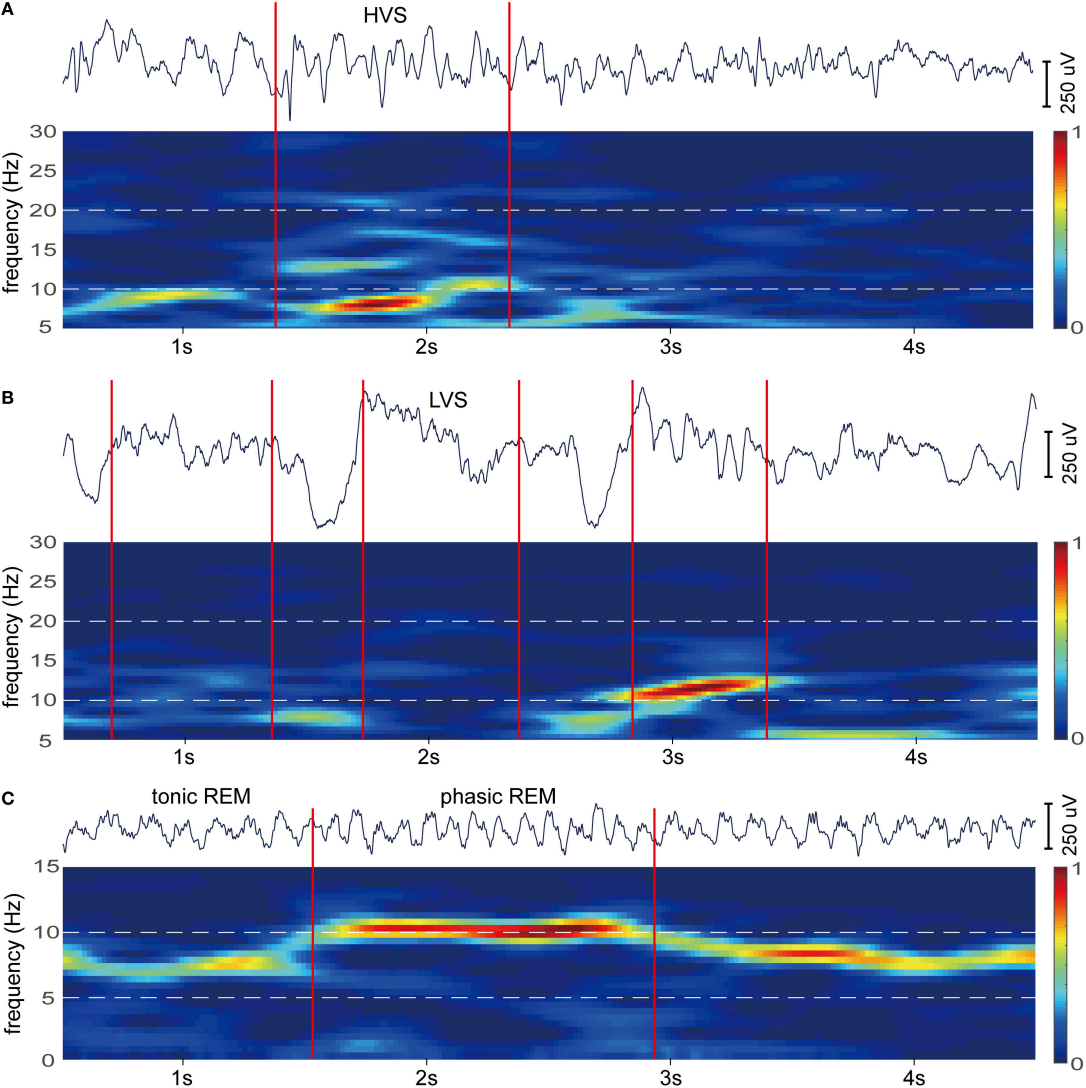
**Example epochs of HVS, LVS, tonic REM, and phasic REM for RSC. (A)** EEG of HVS epoch occur during periods of SWS (top). In the spectrogram of the EEG (bottom), HVS is characterized by a strong peak at 6–9 Hz. **(B)** EEG of LVS epochs occur during periods of SWS (top). In the spectrogram of the EEG (bottom), LVSs correspond to a peak range from 10 to 20 Hz. **(C)** EEG of tonic and phasic REM occur during periods of REMS (top). In the spectrogram of the EEG (bottom), tonic REM is marked by a strong peak around 6 Hz, however, phasic REM is marked around 10 Hz. Red lines indicate the onset of epochs. Spectrograms were calculated with EEGLAB (http://sccn.ucsd.edu/eeglab/; default parameter). Bars are normalized and hot colors reflect high power.

To further confirm the subdivision of spindle and theta oscillations, epochs selected from SWS and REMS were examined with time-frequency analysis. Original EEG activities and their spectrograms of RSC reveal two types of spindles with different waveforms and spectrograms (Figures 6A,B), and two types of theta activities with discrete spectrograms (Figure 6C). Additionally, these diverse oscillation activities happened at separate moments (Figure 6), which further support the frequency band subdivisions of spindle and theta.

## Discussions

The present study used factor analysis to extract the frequency bands across brain regions during WR, SWS, and REMS. We found that frequency band divisions varied both across vigilance states and brain regions. These results suggested that the frequency bands divisions were determined by vigilance states and specific neural mass in distinct brain regions.

### The frequency band divisions of EEG

In both human and rat, two types of REMS can be differentiated, i.e., tonic and phasic REMS, which the latter is characterized by ponto-geniculo-occipital (PGO) waves (pontine waves in rats), increased in the frequency of theta waves, and muscle twitches (Horne, 2000; Karashima et al., 2005; Montgomery et al., 2008). In the present work, obvious theta oscillations with 7 Hz peaks were found across all the brain regions during REMS. However, unlike the customary frequency band division in rats, the theta was further divided into two bands (5–7 and 8–11 Hz) in most regions. These divisions are in line with behavioral evidence of two kinds of REMS (Karashima et al., 2005; Montgomery et al., 2008). In humans, thalamo-cortical network specifically activates during phasic REMS, accompanying with lower alertness compared with tonic REMS (Wehrle et al., 2007). In rats, although both types of REMS are speculated to be involved in mnemonic process (Karashima et al., 2005), recent studies suggested the diverse roles of them in the mnemonic process (Montgomery et al., 2008; Brankack et al., 2012). Thus, the subdivision of theta bands may be more coincident with the brain functional states.

Previous studies have reported that there are two different sleep spindles, i.e., low-frequency and high-voltage spindles (HVSs: 6–10 Hz, primarily 7–8 Hz) and high-frequency and low-voltage spindles (LVSs: 6–20 Hz, primarily 10–20 Hz) (Kandel and Buzsaki, 1997; Johnson et al., 2010), and the former has a larger amplitude (about 3 to 5 times) than the latter (Buzsaki, 1991). Consistent with these findings, we have identified similar two bands (6–10 and 9–21 Hz) with different power spectra during SWS using both factor analysis (Table 4) and time-frequency analysis (Figure 6). Despite that HVSs and LVSs occupy similar or sometimes overlapping frequency bands and share many of the cellular mechanisms (Kandel and Buzsaki, 1997), their functional significances are quite different. LVSs are believed to be correlated with memory consolidation, whereas HVSs are not or even associated with memory deficits (Johnson et al., 2010). Compared with the classical division of the spindle, the exact subdivision of spindle bands may be more meaningful.

### Variation of functional band divisions across brain regions

During SWS, the frequency band divisions of LVSs were different along the anterior-posterior regions, i.e., the “slow” spindles (~16 Hz) that were restricted to frontal regions (PrL and CG) and the “fast” spindles (~21 Hz) that were found in parietal areas (RSC and V2). To the best of our knowledge, no studies related to the spindle activities have distinguished the two spindle components in rats. What's more, from the perspective of the spatial distribution, the current findings are consistent with the results in humans (9–12 and 12–15 Hz for slow and fast spindles, respectively) and cats (12–14 and 14–16 Hz for slow and fast spindles, respectively) (Mölle et al., 2011; Timofeev and Chauvette, 2013). Moreover, these two types of spindles may originate from different sources (Mölle et al., 2011; Timofeev and Chauvette, 2013), and take part in the diverse phases of memory consolidation (Mölle et al., 2011).

During WR, unlike the other regions, functional binding of delta and theta was found in both V2 and TE (Figure 3). In this state, rat hippocampus shows large irregular activity (Vanderwolf and Robinson, 1981), moreover, theta activity can originate in RSC independent from hippocampus (Talk et al., 2004; Young and McNaughton, 2009). It is reasonable to infer that the theta oscillation in WR might originate from RSC (note that this speculation doesn't exclude other potential generators). Furthermore, RSC tightly connected to the anterior thalamic nuclei (Vann et al., 2009; Dalrymple-Alford et al., 2015), and theta-like unit activity can be found in RSC and anterior thalamic nuclei simultaneously (Talk et al., 2004). Ultimately, the co-variation of delta and theta in V2 and TE might reflect a coordinated activities of the thalamus and RSC. Functionally, this co-variation might facilitate the information integration across sensory regions, supporting the past remembering and/or future imaging in this behavior state (Vann et al., 2009; Mednick et al., 2011).

For REMS, the phasic RMES theta co-varied with frequencies from 13–15 to 16–18 Hz across regions (except TE). Because phase synchronizations may occur for harmonic oscillation (Pletzer et al., 2010), and the 14 Hz peaks (the second harmonic of 7 Hz) were consistently found across regions. These co-variations of the phasic REMS theta bands with additional frequency bands might only indicate the effect of harmonic frequency relationships rather than the functional oscillation binding. There is no clear explanation for the co-variations in TE both during SWS and REMS, but it cannot be attributed to probable artifacts, since both SWS and REMS have lower muscle activities compared with WR. As for PrL, the HVSs were not identified in this region. However, the same frequency division (7–14 Hz for spindles) was also found in the previous study (Siapas and Wilson, 1998), reflecting probably PrL has not been involved in HVS activity.

Compared with other brain regions, the theta division of RSC perfectly fit the power spectra during WR (Figure 3). Interestingly, the RSC may be the hypothetical generator (discussed above) of this theta activity in WR. Similarly, hippocampal theta activity in rodents can be recorded in other neocortex (Petsche and Stumpf, 1960; Winson, 1974; Bland and Whishaw, 1976). In the current study, band divisions of structures (RSC and V2) above hippocampus have better fit with power spectra than others during REMS. These findings indicate that the results of factor analysis may be affected by the constructions and locations related to the underlying generators to some extent, for which the difference of the division may be caused by the different signal-to-noise ratios. However, it should be noted that this speculation cannot exclude the influences of other potential generators. Actually, many independent theta generators have been observed in rats (Bilkey and Heinemann, 1999; Kahana et al., 2001; Seidenbecher et al., 2003; Young and McNaughton, 2009). Nevertheless, RSC and hippocampus may dominate the theta activities during WR and REMS, respectively. Therefore, the results of factor analysis might be influenced by the potential generators, and spatial factor should be considered in frequency band divisions when using factor analysis in rats.

The specific frequency bands identified by factor analysis across brain regions and states reflect the potential generators and functional significances. Therefore, it is worth noting that frequency band divisions based on distributed brain regions are meaningful for brain functional studies.

### Comparison with a previous study

Based on our knowledge, there is one closely related study in rats (Corsi-Cabrera et al., 2001). The present study has three obvious differences from the previous one. First of all, during WR, the delta activities were identified only in the present work. This is quite possibly caused by the difference between wakefulness and wakeful rest, and the delta activity is consistent with the findings in Young and McNaughton (2009). Secondly, during REMS, the theta oscillations were divided into two bands in this study. This difference may be due to the different data samples. In the work of Corsi-Cabrera et al., the data size is relatively small, and the phasic REMS occupy only approximately 5% of the total REMS (Montgomery et al., 2008). Finally, despite the similarity of statistical changes of the power spectra (Corsi-Cabrera et al., 2001), however, in the current study, we found a significant decrease of power (range from 10 to 28 Hz) during REMS compared with WR in PrL, CG, and RSC. These decreased power are supported by the brain imaging study in humans, in which a lack of increased blood flow in the homologous brain regions during REMS (Maquet et al., 1996). Moreover, these homologous brain regions in humans densely connected with the medial pulvinar nucleus (PuM) of thalamus, and decreased power (beta and gamma) was also found in PuM during REMS (Magnin et al., 2004). Interestingly, unlike in PrL, CG, and RSC, this decrease was not found in V2 and TE (also in results of Corsi-Cabrera et al.). It is possible that PrL, CG, and RSC are all association cortex with extensive connections (Vogt and Miller, 1983; Conde et al., 1990, 1995; Vann et al., 2009; Vann, 2013). This situation could reflect an incomplete interaction between brain regions, a phenomenon resulting in a distracting state of REMS (Horne, 2000).

Although our method used in the current study is similar to the former one, we further considered the spatial factors on the frequency band divisions and used a bigger data size, with which the results observed here are also interesting. Especially, in the present work with a bigger data size, we found the subdivision of spindle and theta activities, which were not found in the former one. Furthermore, we also found functional relevant frequencies (delta and theta) co-vary in some brain regions and changed power of some frequency bands limited in other brain regions. We believe these findings could provide us some new insights into brain oscillations across brain regions and vigilance states.

### Limitations

Although there are numerous advantages in our current study using factor analysis with considerations of spatial factors, we cannot exclude several limitations. Firstly, the results of factor analysis are based on power spectra, it should be noted that the power spectra are greatly affected by reference electrode. However, this limitation should not affect the conclusions since (1) the absolute mean power of cerebellar activity is several folds lower than that at the cerebral level, cerebellum as referenced is more like a rest reference at infinity than other cortexes (Culic et al., 2003) and is widely used in the rat electrophysiological study; (2) the power spectra in current work are similar to that in the former study, in which the reference electrode was not placed above the cerebellum (Corsi-Cabrera et al., 2001); (3) in this work, factor analysis was also conducted with average reference, and the results were similar (data not shown).

Secondly, present results were obtained with two recording technologies (ECoG and LFP), which allows for different spatial resolutions. However, this limitation should not affect the conclusions since (1) each brain region was used only one recording technology and independently conducted with factor analysis; (2) differences of band divisions widely distributed across regions, rather than limited between two different recording technologies.

Finally, it should be pointed out that EEG properties differ along the left-right axes of the rat brain (Fang et al., 2010). Whereas, lateralization of frequency band divisions was not taken into consideration in the current work, this question will be addressed in future work.

### Summary

In this study, we used factor analysis on averaged EEG power spectra derived from multiple brain regions across vigilance states in rats. We found that the frequency band divisions changed both across states and brain regions. These findings not only provide more precise frequency band divisions, but also promote us to consider the impacts of spatial factors on the frequency band divisions.

## Author contributions

DY, YX, and WJ: conceived and designed the study. WJ, YW, and MX: conducted the experiments. WJ, MC, DG, and GF: performed data analysis and prepared the draft manuscript. WJ, DY, and GF: reviewed data interpretation. DY and YX: edited and approved final manuscript.

## Funding

This work was supported by National Natural Science Foundation of China No. 81371636, No. 81330032, No. 61527815, No. 91232725, No. 81571770, No. 31372217, and the 111 project (B12027).

### Conflict of interest statement

The authors declare that the research was conducted in the absence of any commercial or financial relationships that could be construed as a potential conflict of interest.

## Supplementary material

Figure S1 **Histological tests show the electrode positions. (A)** Electrode position of CG. **(B)** Electrode position of PrL. The arrows denote the recording tips.

Figure S2 **Correlation coefficient between factors (frequency bands) across states and brain regions**. The # represent the correlation coefficient cannot reach the significant level (*p* < 0.05). F, factor.

## References

[B1] Babiloni C. Squitti R. Del Percio C. Cassetta E. Ventriglia M. C. Ferreri F.. (2007). Free copper and resting temporal EEG rhythms correlate across healthy, mild cognitive impairment, and Alzheimer's disease subjects. Clin. Neurophysiol. 118, 1244–1260. 10.1016/j.clinph.2007.03.016 17462944

[B2] Basar E. Basar-Eroglu C. Karakas S. Schürmann M. (2001). Gamma, alpha, delta, and theta oscillations govern cognitive processes. Int. J. Psychophysiol. 39, 241–248. 10.1016/S0167-8760(00)00145-8 11163901

[B3] Basar E. Güntekin B. (2008). A review of brain oscillations in cognitive disorders and the role of neurotransmitters. Brain Res. 1235, 172–193. 10.1016/j.brainres.2008.06.103 18640103

[B4] Bilkey D. K. Heinemann U. (1999). Intrinsic theta-frequency membrane potential oscillations in layer III/V perirhinal cortex neurons of the rat. Hippocampus 9, 510–518. 10560921 10.1002/(SICI)1098-1063(1999)9:5<510::AID-HIPO4>3.0.CO;2-9

[B5] Bland B. H. Whishaw I. Q. (1976). Generators and topography of hippocampal theta (RSA) in the anaesthetized and freely moving rat. Brain Res. 118, 259–280. 10.1016/0006-8993(76)90711-3 1000290

[B6] Brankack J. Scheffzük C. Kukushka V. I. Vyssotski A. L. Tort A. B. Draguhn A. (2012). Distinct features of fast oscillations in phasic and tonic rapid eye movement sleep. J. Sleep Res. 21, 630–633. 10.1111/j.1365-2869.2012.01037.x 22812730

[B7] Brown R. E. Basheer R. McKenna J. T. Strecker R. E. McCarley R. W. (2012). Control of sleep and wakefulness. Physiol. Rev. 92, 1087–1187. 10.1152/physrev.00032.2011 22811426 PMC3621793

[B8] Buzsaki G. (1991). The thalamic clock: emergent network properties. Neuroscience 41, 351–364. 10.1016/0306-4522(91)90332-I 1870695

[B9] Buzsáki G. (2006). Rhythms of the Brain. New York, NY: Oxford University Press.

[B10] Buzsaki G. Draguhn A. (2004). Neuronal oscillations in cortical networks. Science 304, 1926–1929. 10.1126/science.1099745 15218136

[B11] Cohen J. (1992). A power primer. Psychol. Bull. 112, 155–159. 10.1037/0033-2909.112.1.155 19565683

[B12] Conde F. Audinat E. Maire-Lepoivre E. Crepel F. (1990). Afferent connections of the medial frontal cortex of the rat. A study using retrograde transport of fluorescent dyes. I. Thalamic afferents. Brain Res. Bull. 24, 341–354. 10.1016/0361-9230(90)90088-H 2337814

[B13] Conde F. Maire-Lepoivre E. Audinat E. Crepel F. (1995). Afferent connections of the medial frontal cortex of the rat. II. Cortical and subcortical afferents. J. Comp. Neurol. 352, 567–593. 10.1002/cne.903520407 7722001

[B14] Corsi-Cabrera M. Guevara M. A. Del Rio-Portilla Y. Arce C. Villanueva-Hernandez Y. (2000). EEG bands during wakefulness, slow-wave and paradoxical sleep as a result of principal component analysis in man. Sleep 23, 738–744. 11007440

[B15] Corsi-Cabrera M. Perez-Garci E. Del Rio-Portilla Y. Ugalde E. Guevara M. A. (2001). EEG bands during wakefulness, slow-wave, and paradoxical sleep as a result of principal component analysis in the rat. Sleep 24, 374–380. 11403521 10.1093/sleep/24.4.374

[B16] Culic M. Grbic G. Martac Blanusa L. Spasic S. Jankovic B. Rankovic R. (2003). Slow and fast oscillations in the activity of parietal cortex after brain injury, in From Basic Motor Control to Functional Recovery III, ed Gantchev N. (Sofia: Kliment Ohridski University Press), 41–45.

[B17] Dalrymple-Alford J. C. Harland B. Loukavenko E. A. Perry B. Mercer S. Collings D. A.. (2015). Anterior thalamic nuclei lesions and recovery of function: relevance to cognitive thalamus. Neurosci. Biobehav. Rev. 54, 145–160. 10.1016/j.neubiorev.2014.12.007 25637779

[B18] Dossi R. C. Nunez A. Steriade M. (1992). Electrophysiology of a slow (0.5-4 Hz) intrinsic oscillation of cat thalamocortical neurones *in vivo*. J. Physiol. 447, 215–234. 10.1113/jphysiol.1992.sp018999 1593448 PMC1176033

[B19] Engel A. K. Fries P. Singer W. (2001). Dynamic predictions: oscillations and synchrony in top-down processing. Nat. Rev. Neurosci. 2, 704–716. 10.1038/35094565 11584308

[B20] Eschenko O. Mölle M. Born J. Sara S. J. (2006). Elevated sleep spindle density after learning or after retrieval in rats. J. Neurosci. 26, 12914–12920. 10.1523/JNEUROSCI.3175-06.2006 17167082 PMC6674950

[B21] Fang G. Chen Q. Cui J. Tang Y. (2012). Electroencephalogram bands modulated by vigilance states in an anuran species: a factor analytic approach. J. Comp. Physiol. A. Neuroethol. Sens. Neural. Behav. Physiol. 198, 119–127. 10.1007/s00359-011-0693-y 22045113

[B22] Fang G. Xia Y. Lai Y. You Z. Yao D. (2010). Long-range correlations of different EEG derivations in rats: sleep stage-dependent generators may play a key role. Physiol. Meas. 31, 795–808. 10.1088/0967-3334/31/6/005 20453294

[B23] Grasing K. Szeto H. (1992). Diurnal variation in continuous measures of the rat EEG power spectra. Physiol. Behav. 51, 249–254. 10.1016/0031-9384(92)90138-R 1557436

[B24] Horne J. A. (2000). REM sleep - by default? Neurosci. Biobehav. Rev. 24, 777–797. 10.1016/S0149-7634(00)00037-3 11118606

[B25] Johnson L. A. Euston D. R. Tatsuno M. McNaughton B. L. (2010). Stored-Trace reactivation in rat prefrontal cortex is correlated with down-to-up state fluctuation density. J. Neurosci. 30, 2650–2661. 10.1523/JNEUROSCI.1617-09.2010 20164349 PMC2917239

[B26] Kahana M. J. Seelig D. Madsen J. R. (2001). Theta returns. Curr. Opin. Neurobiol. 11, 739–744. 10.1016/S0959-4388(01)00278-1 11741027

[B27] Kandel A. Buzsaki G. (1997). Cellular-synaptic generation of sleep spindles, spike-and-wave discharges, and evoked thalamocortical responses in the neocortex of the rat. J. Neurosci. 17, 6783–6797. 9254689 10.1523/JNEUROSCI.17-17-06783.1997 PMC6573130

[B28] Karashima A. Nakao M. Katayama N. Honda K. (2005). Instantaneous acceleration and amplification of hippocampal theta wave coincident with phasic pontine activities during REM sleep. Brain Res. 1051, 50–56. 10.1016/j.brainres.2005.05.055 15982642

[B29] Klimesch W. (1999). EEG alpha and theta oscillations reflect cognitive and memory performance: a review and analysis. Brain Res. Rev. 29, 169–195. 10.1016/S0165-0173(98)00056-3 10209231

[B30] Lancel M. Kerkhof G. A. (1989). Effects of repeated sleep deprivation in the dark- or light-period on sleep in rats. Physiol. Behav. 45, 289–297. 10.1016/0031-9384(89)90130-3 2756014

[B31] Leung L. W. Yim C. Y. (1991). Intrinsic membrane potential oscillations in hippocampal neurons *in vitro*. Brain Res. 553, 261–274. 10.1016/0006-8993(91)90834-I 1718544

[B32] Lomas T. Ivtzan I. Fu C. H. (2015). A systematic review of the neurophysiology of mindfulness on EEG oscillations. Neurosci. Biobehav. Rev. 57, 401–410. 10.1016/j.neubiorev.2015.09.018 26441373

[B33] Magnin M. Bastuji H. Garcia-Larrea L. Mauguiere F. (2004). Human thalamic medial pulvinar nucleus is not activated during paradoxical sleep. Cereb. Cortex 14, 858–862. 10.1093/cercor/bhh044 15054059

[B34] Maquet P. Péters J. Aerts J. Delfiore G. Degueldre C. Luxen A.. (1996). Functional neuroanatomy of human rapid-eye-movement sleep and dreaming. Nature 383, 163–166. 10.1038/383163a0 8774879

[B35] Mednick S. C. Cai D. J. Shuman T. Anagnostaras S. Wixted J. T. (2011). An opportunistic theory of cellular and systems consolidation. Trends Neurosci. 34, 504–514. 10.1016/j.tins.2011.06.003 21742389 PMC3183157

[B36] Meeren H. K. van Cappellen van Walsum A. M. van Luijtelaar E. L. Coenen A. M. (2001). Auditory evoked potentials from auditory cortex, medial geniculate nucleus, and inferior colliculus during sleep-wake states and spike-wave discharges in the WAG/Rij rat. Brain Res. 898, 321–331. 10.1016/S0006-8993(01)02209-0 11306019

[B37] Mitchell D. J. McNaughton N. Flanagan D. Kirk I. J. (2008). Frontal-midline theta from the perspective of hippocampal “theta.” Prog. Neurobiol. 86, 156–185. 10.1016/j.pneurobio.2008.09.005 18824212

[B38] Mölle M. Bergmann T. O. Marshall L. Born J. (2011). Fast and Slow Spindles during the sleep slow oscillation: disparate coalescence and engagement in memory processing. Sleep 34, 1411–U1162. 10.5665/sleep.1290 21966073 PMC3174843

[B39] Mölle M. Yeshenko O. Marshall L. Sara S. J. Born J. (2006). Hippocampal sharp wave-ripples linked to slow oscillations in rat slow-wave sleep. J. Neurophysiol. 96, 62–70. 10.1152/jn.00014.2006 16611848

[B40] Montgomery S. M. Sirota A. Buzsaki G. (2008). Theta and gamma coordination of hippocampal networks during waking and rapid eye movement sleep. J. Neurosci. 28, 6731–6741. 10.1523/JNEUROSCI.1227-08.2008 18579747 PMC2596978

[B41] Nicolelis M. A. Lin S.-C. Gervasoni D. (2008). Defining Global Brain States Using Multielectrode Field Potential Recordings. Boca Raton, FL: CRC Press. 21204450

[B42] Nunez A. Garcia-Austt E. Buno W. Jr. (1987). Intracellular theta-rhythm generation in identified hippocampal pyramids. Brain Res. 416, 289–300. 10.1016/0006-8993(87)90909-7 3620962

[B43] Nunez P. L. Cutillo B. A. (1995). Neocortical Dynamics and Human EEG Rhythms. New York, NY: Oxford University Press.

[B44] Paxinos G. Watson C. (2005). The Rat Brain in Stereotaxic Coordinates. Burlington, VT: Elsevier Academic Press.

[B45] Petsche H. Stumpf C. (1960). Topographic and toposcopic study of origin and spread of the regular synchronized arousal pattern in the rabbit. Electroen. Clin. Neuro. 12, 589–600. 10.1016/0013-4694(60)90101-2 14432410

[B46] Peyrache A. Battaglia F. P. Destexhe A. (2011). Inhibition recruitment in prefrontal cortex during sleep spindles and gating of hippocampal inputs. Proc. Natl. Acad. Sci. U.S.A. 108, 17207–17212. 10.1073/pnas.1103612108 21949372 PMC3193185

[B47] Pletzer B. Kerschbaum H. Klimesch W. (2010). When frequencies never synchronize: the golden mean and the resting EEG. Brain Res. 1335, 91–102. 10.1016/j.brainres.2010.03.074 20350536

[B48] Riboni L. Luna F. Nunez-Duran H. (1991). A fast staining method for CNS slices. J. Neurosci. Methods 38, 239–241. 10.1016/0165-0270(91)90174-X 1723778

[B49] Seidenbecher T. Laxmi T. R. Stork O. Pape H. C. (2003). Amygdalar and hippocampal theta rhythm synchronization during fear memory retrieval. Science 301, 846–850. 10.1126/science.1085818 12907806

[B50] Siapas A. G. Wilson M. A. (1998). Coordinated interactions between hippocampal ripples and cortical spindles during slow-wave sleep. Neuron 21, 1123–1128. 10.1016/S0896-6273(00)80629-7 9856467

[B51] Sirota A. Csicsvari J. Buhl D. Buzsaki G. (2003). Communication between neocortex and hippocampus during sleep in rodents. Proc. Natl. Acad. Sci. U.S.A. 100, 2065–2069. 10.1073/pnas.0437938100 12576550 PMC149959

[B52] Steriade M. McCormick D. A. Sejnowski T. J. (1993). Thalamocortical oscillations in the sleeping and aroused brain. Science 262, 679–685. 10.1126/science.8235588 8235588

[B53] Talk A. Kang E. Gabriel M. (2004). Independent generation of theta rhythm in the hippocampus and posterior cingulate cortex. Brain Res. 1015, 15–24. 10.1016/j.brainres.2004.04.051 15223362

[B54] Thut G. Miniussi C. (2009). New insights into rhythmic brain activity from TMS-EEG studies. Trends Cogn. Sci. 13, 182–189. 10.1016/j.tics.2009.01.004 19286414

[B55] Timofeev I. Chauvette S. (2013). The spindles: are they still thalamic? Sleep 36, 825–826. 10.5665/sleep.2702 23729924 PMC3649824

[B56] Trampus M. Conti A. Marzanatti M. Monopoli A. Ongini E. (1990). Effects of the enkephalinase inhibitor SCH 34826 on the sleep-waking cycle and EEG activity in the rat. Neuropharmacology 29, 199–205. 10.1016/0028-3908(90)90002-9 2325830

[B57] Vanderwolf C. Robinson T. (1981). Reticulo-cortical activity and behavior: a critique of the arousal theory and a new synthesis. Behav. Brain Sci. 4, 459–476. 10.1017/S0140525X00009869

[B58] Vann S. D. (2013). Dismantling the Papez circuit for memory in rats. Elife 2:e00736. 10.7554/eLife.00736 23805381 PMC3691571

[B59] Vann S. D. Aggleton J. P. Maguire E. A. (2009). What does the retrosplenial cortex do? Nat. Rev. Neurosci. 10, 792–802. 10.1038/nrn2733 19812579

[B60] Vogt B. A. Miller M. W. (1983). Cortical connections between rat cingulate cortex and visual, motor, and postsubicular cortices. J. Comp. Neurol. 216, 192–210. 10.1002/cne.902160207 6863602

[B61] Wehrle R. Kaufmann C. Wetter T. C. Holsboer F. Auer D. P. Pollmacher T.. (2007). Functional microstates within human REM sleep: first evidence from fMRI of a thalamocortical network specific for phasic REM periods. Eur. J. Neurosci. 25, 863–871. 10.1111/j.1460-9568.2007.05314.x 17328781

[B62] Winson J. (1974). Patterns of hippocampal theta rhythm in the freely moving rat. Electroen. Clin. Neuro. 36, 291–301. 10.1016/0013-4694(74)90171-0 4130608

[B63] Young C. K. McNaughton N. (2009). Coupling of theta oscillations between anterior and posterior midline cortex and with the hippocampus in freely behaving rats. Cereb. Cortex 19, 24–40. 10.1093/cercor/bhn055 18453538

