# Supplementary figures and images for: EEG Bands of Wakeful Rest, Slow-Wave and Rapid-Eye-Movement Sleep at Different Brain Areas in Rats
Source: Front Comput Neurosci. 2016 Aug 3;10:79. doi: 10.3389/fncom.2016.00079 (PMC4971061; doi:10.3389/fncom.2016.00079)

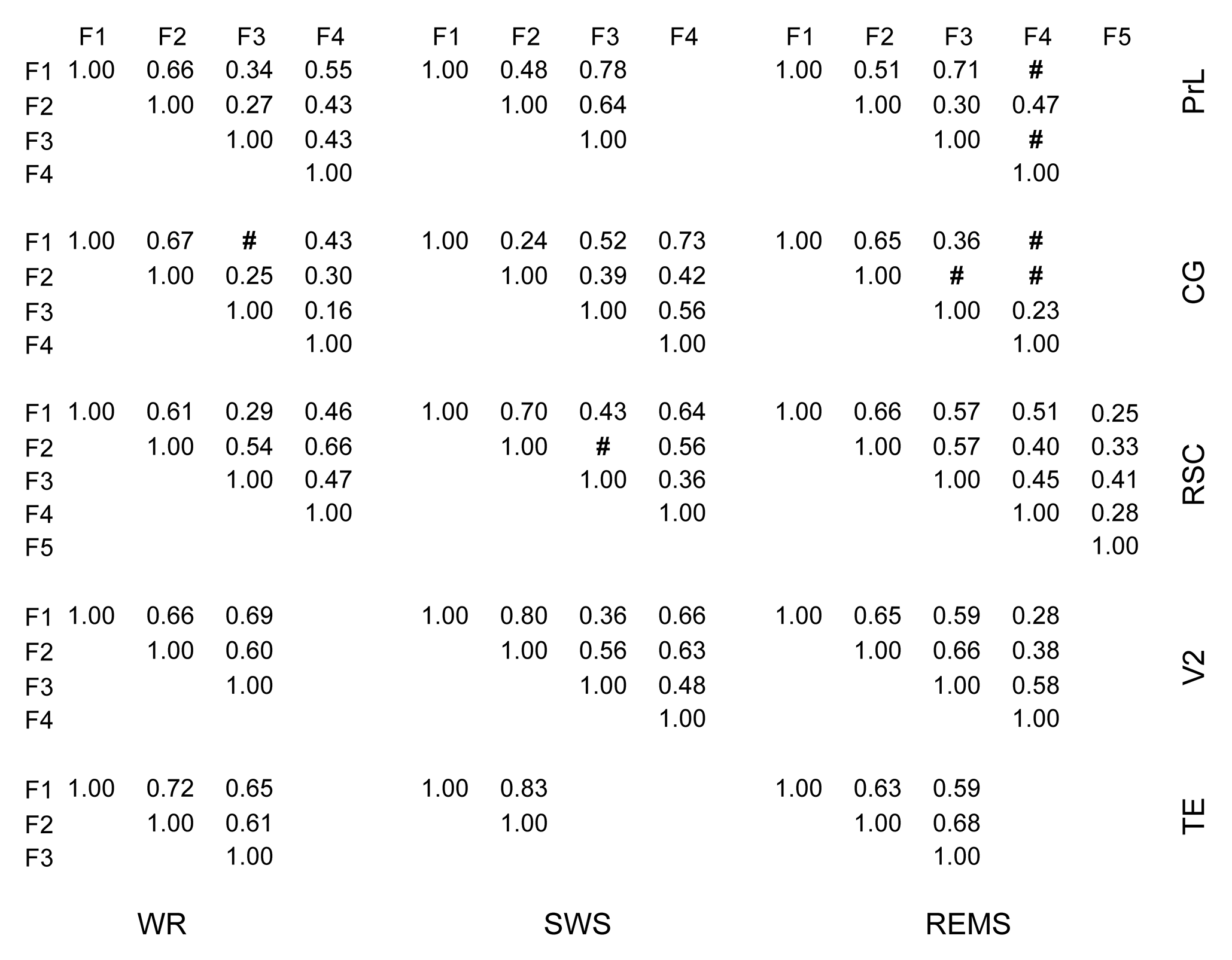

Supplement: Figure S2 — Correlation coefficient between factors (frequency bands) across states and brain regions. The # represent the correlation coefficient cannot reach the significant level (p < 0.05). F, factor. [file Image2.TIF]
